# Palmitoleic acid prevents palmitic acid-induced macrophage activation and consequent p38 MAPK-mediated skeletal muscle insulin resistance

**DOI:** 10.1016/j.mce.2014.06.010

**Published:** 2014-08-05

**Authors:** Nicola A. Talbot, Caroline P. Wheeler-Jones, Mark E. Cleasby

**Affiliations:** Department of Comparative Biomedical Sciences, Royal Veterinary College, University of London, Royal College Street, London NW1 0TU, UK

**Keywords:** ABAF, anti-bacterial, anti-fungal, ANOVA, analysis of variance, AS160, Akt substrate of 160 kDa, BSA, bovine serum albumin, CM, conditioned medium, CXCL2, Chemokine (C-X-C motif) ligand 2, DMEM, Dulbecco’s modified Eagle's medium, DMSO, dimethylsulphoxide, ERK, extracellular signal-related kinase, FA, fatty acid, FBS, foetal bovine serum, GLUT, glucose transporter, GSK, glycogen synthase kinase, IKK, inhibitor κ kinase, IκBα, inhibitor κBα, IL, interleukin, iNOS, inducible nitric oxide synthase, IR, insulin resistance, IRS1, insulin receptor substrate-1, JNK, C-jun n-terminal kinase, LPS, lipopolysaccharide, mac, macrophage, MAPK, mitogen-activated protein kinase, MCP1, monocyte chemoattractant protein, NFκB, nuclear factor-κB, PI3K, phosphoinositol 3-kinase, palm, palmitate, PBS, phosphate-buffered saline, PKC, protein kinase C, PMA, phorbol myristate acetate, RIPA, radioimmunoprecipitation, SDS-PAGE, sodium dodecyl sulphate, polyacrylamide gel electrophoresis, SFA, saturated fatty acid, siRNA, small interfering RNA, T2D, type 2 diabetes, TLR, Toll-like Receptor, TNFα, tumour necrosis factor-α, UFA, unsaturated fatty acid, Fatty acid, Tumour necrosis factor-α, p38 Mitogen-activated protein kinase, Insulin resistance, Skeletal muscle, Macrophage

## Abstract

Obesity and saturated fatty acid (SFA) treatment are both associated with skeletal muscle insulin resistance (IR) and increased macrophage infiltration. However, the relative effects of SFA and unsaturated fatty acid (UFA)-activated macrophages on muscle are unknown. Here, macrophages were treated with palmitic acid, palmitoleic acid or both and the effects of the conditioned medium (CM) on C2C12 myotubes investigated. CM from palmitic acid-treated J774s (palm-mac-CM) impaired insulin signalling and insulin-stimulated glycogen synthesis, reduced Inhibitor κBα and increased phosphorylation of p38 mitogen-activated protein kinase (MAPK) and c-Jun N-terminal kinase in myotubes. p38 MAPK inhibition or siRNA partially ameliorated these defects, as did addition of tumour necrosis factor-α blocking antibody to the CM. Macrophages incubated with both FAs generated CM that did not induce IR, while palmitoleic acid-mac-CM alone was insulin sensitising. Thus UFAs may improve muscle insulin sensitivity and counteract SFA-mediated IR through an effect on macrophage activation.

## Highlights

• Palmitate-treated macrophage-conditioned medium causes myotube insulin resistance.
• This involves activation of myotube p38 mitogen activated protein kinase.
• Conditioned medium effects are mediated by tumour necrosis factor-α.
• These effects are prevented by addition of palmitoleate.
• Palmitoleate treatment of macrophages is insulin sensitising for myotubes.

## 1 Introduction

Insulin resistance (IR) in skeletal muscle develops in advance of type 2 diabetes (T2D) in humans and typically occurs alongside obesity and elevated plasma lipid and fatty acid (FA) levels. It is well-established that accumulation of lipid derivatives in skeletal muscle results in IR (Turner et al., 2014) and that saturated (SFAs) impair insulin-stimulated glucose disposal through inhibition of the phosphoinositol 3-kinase (PI3K)/protein kinase B (Akt) signalling pathway (Cazzolli et al., 2001, Dimopoulos et al., 2006, Griffin et al., 1999, Hirabara et al., 2010, Hommelberg et al., 2011, Kadotani et al., 2009, Storlien et al., 1991), while some unsaturated fatty acids (UFAs) have been shown to have insulin sensitising effects (Kadotani et al., 2009; Tardif et al., 2011) and to be capable of negating the deleterious effects of SFAs (Coll et al., 2008, Dimopoulos et al., 2006, Gao et al., 2009, Storlien et al., 1991). Obesity represents a subclinical inflammatory state, evidenced by an association with markers of systemic inflammation (Festa et al., 2002) and increased infiltration of innate immune cells, including macrophages, into obese adipose tissue (Cancello et al., 2005, Elgazar-Carmon et al., 2008, Weisberg et al., 2003). The normal resident macrophage population in lean adipose tissue is modified in obesity. Resident adipose macrophages in lean individuals are predominantly activated towards an M2-like phenotype and release anti-inflammatory cytokines, such as interleukin (IL)-10, which are insulin sensitising (Lumeng et al., 2007a). However, in obesity macrophage phenotype switches towards a more pro-inflammatory M1-like subtype and/or they release chemokines that recruit additional macrophages (Lumeng et al., 2007a, Lumeng et al., 2007b, Prieur et al., 2011).

It is as yet unclear whether FAs or other lipid derivatives are responsible for direct activation of macrophages or if macrophages are activated by signals from other tissues or cells, given the extensive cross-talk that occurs between adipocytes and infiltrating inflammatory cells (Lumeng et al., 2007a, Lumeng et al., 2007b, Prieur et al., 2011). However, recent evidence suggests that SFAs may bind Toll-like-receptors (TLR) on macrophages (Hwang, 2001, Lee et al., 2001, Nguyen et al., 2007), leading to activation of inflammatory pathways such as the classical nuclear factor-κB (NF-κB) and c-jun n-terminal kinase (JNK) pathways and elevated production of pro-inflammatory cytokines (Haversen et al., 2009, Hwang, 2001, Samokhvalov et al., 2008, Shi et al., 2006), while this effect can be blocked by UFAs (Haversen et al., 2009, Lee et al., 2004). A widely demonstrated mechanism whereby cytokines and SFAs may induce IR is via activation of stress kinases and inhibitory serine phosphorylation of insulin receptor substrate-1 (IRS-1) (McCall et al., 2010, Solinas et al., 2006), a key adaptor protein activating phosphoinositol 3-kinase (PI3K) signalling and thus insulin-stimulated glucose disposal in muscle. These kinases include the mitogen-activated protein kinases (MAPKs) c-jun n-terminal kinase (JNK), p38 MAPK and Inhibitor κB kinase, upstream of NFκB and indeed the deletion of JNK-1 (Hirosumi et al., 2002) and IκB kinase (IKK) (Yuan et al., 2001) prevented high-fat diet (HFD) – induced IR in mice.

Whereas some studies have demonstrated that macrophages accumulate in tissues other than adipose during obesity, including skeletal muscle ((Fink et al., 2013, Hevener et al., 2007, Nguyen et al., 2007); NAT, CWJ and MEC, unpublished data) little attention has been paid to the potential impact of this upon muscle insulin sensitivity. However, a recent study suggested that macrophages in muscle from obese individuals may also contribute to the increase in pro-inflammatory cytokine release and thus muscle IR (Varma et al., 2009). Furthermore, after incubation with the SFA palmitic acid, pro-inflammatory pathways are activated in macrophages, leading to IR in adipocytes through a paracrine loop involving tumour necrosis factor (TNF)α (Suganami et al., 2005), which has been repeatedly shown to induce IR in muscle cells *in vitro* and *in vivo* (de Alvaro et al., 2004, Hotamisligil et al., 1994, Liang et al., 2008, Plomgaard et al., 2005, Uysal et al., 1997). The effects of TNFα may be mediated through p38 MAPK, as inhibition or silencing of this kinase *in vitro* ameliorated TNFα-induced skeletal muscle IR (de Alvaro et al., 2004).

Recently, palmitic acid-treated macrophages were shown to generate conditioned medium (CM) that reduced glucose uptake and PI3K signalling and increased inflammatory signalling in GLUT4-overexpressing L6 myoblasts (Samokhvalov et al., 2008), an effect that was mediated through induction of protein kinase C (PKC) *θ* and *ε* isoforms (Kewalramani et al., 2011). In addition, CM from FA-treated macrophages caused IR in L6 myotubes that was TLR2/4-dependent (Nguyen et al., 2007). Conversely, CM from palmitic acid-treated myoblasts was capable of causing a pro-inflammatory switch in macrophage phenotype (Pillon et al., 2012). However, it is unclear whether UFAs might be able to alleviate these effects. Here, we aimed to further interrogate the mechanisms involved in the impairment of insulin sensitivity in differentiated skeletal muscle cells generated by CM derived from SFA-treated macrophages and to establish whether UFA treatment would alleviate these effects.

## 2 Materials and methods

### 2.1 Materials

General reagents were from Sigma–Aldrich (Gillingham, Dorset, UK), cell culture media from Gibco (Life Technologies, Paisley, UK) and recombinant TNFα from eBiosciences (Hatfield, UK). pY612-IRS1 antibody was from Biosource International (Camarillo, CA, USA), total IRS-1 and total glycogen synthase kinase (GSK) 3β antibodies from Millipore (Billerica, MA, USA), β-actin antibody from Sigma and all others from Cell Signaling Technology (Beverley, MA, USA).

### 2.2 Cell culture

C2C12 myoblasts and J774 macrophages were cultured in DMEM containing 4.5 mM glucose, 10% foetal bovine serum (FBS) and 1% antibiotic anti-fungal (ABAF) mixture. Before study, differentiation of myoblasts into myotubes was achieved by switching to DMEM containing 2% horse serum for 5 days. Macrophages were treated with 200 ng/ml phorbol myristate acetate (PMA) for 3 days before use (Karten et al., 1999). Macrophage treatment medium was generated by coupling DMEM containing 10% FBS, 1% ABAF and 2% bovine serum albumin (BSA) with 0.75 mM palmitic acid (SFA), 0.75 mM palmitoleic acid (UFA, chosen because of its identical acyl chain length), a combination of both, or 10 ng/ml of lipopolysaccharide (LPS) as positive control. This was added to J774 cells for 8 h, before being aspirated and the cells washed in PBS x3. Absence of carry-over of FAs into the CM was confirmed by measurement using a kit (Wako Chemicals, Neuss, Germany). Fresh DMEM was then added for 16 h and the CM generated transferred to C2C12 myotubes for a further 16 h. Myotubes were then serum-starved for 2 h and selected wells stimulated with 100nM insulin (Novo Nordisk, Crawley, UK) prior to measurement of glycogen synthesis or lysis and western blotting.

### 2.3 Use of inhibitors

Where pharmacological inhibitors were used, myotubes were pre-treated for 1 h with 1 μM SB203580 and 0.1 μM BIRB796, 1 μM JNK V inhibitor, or vehicle (DMSO), before being treated with CM containing the same substances for 16 h. Where siRNA was employed, C2C12s were transfected with 50 nM nonsense or p38α MAPK-targeting siRNA pool using Dharmafect 3 (Dharmacon, Fisher Scientific, Loughborough, UK) on day 2 of differentiation and then left for 72 h before treatment with CM. Where TNFα blockade was undertaken, half of the CM for each treatment group contained 10 μg/ml of blocking antibody (eBioscience, Hatfield, UK), added before and during myotube incubation with control, palmitic acid or LPS-treated macrophage-CM for 16 h.

### 2.4 SDS–PAGE and immunoblotting

Myotubes were lysed in radioimmunoprecipitation assay (RIPA) buffer, homogenised using an Ultra-Turrax (IKA; Staufen, Germany) and denatured in Laemmli buffer for 10 min at 65 °C. Proteins were resolved by SDS–PAGE, electro-transferred and immunoblotted as previously described (Patel et al., 2012). Specific bands were detected using chemoluminescence (Western Lightning Plus, Perkin Elmer) on Fuji Super RX film (Bedford, UK), scanned and quantified using Quantity One software (BioRad Laboratories, Hemel Hempstead, UK). Loading controls of corresponding total protein immunoreactivity or β-actin were utilised. All treatment groups were represented on each blot on which bands were quantified.

### 2.5 Real-time PCR analysis

Macrophages were collected in Trizol (Invitrogen, Paisley, UK) and homogenised using the Ultra-Turrax. Total RNA was extracted as per the manufacturer’s instructions and resuspended in nuclease-free water. RNA concentration was determined using a Nanodrop 1000 (Wilmington, DE) and integrity confirmed by visualisation of rRNA bands after agarose gel electrophoresis. RNA preparations were DNAse-digested and cDNA was generated using an Omniscript kit (Qiagen, Manchester, UK). Real-time PCR analysis was performed using Fast Start SYBR Green reagent (Roche Diagnostics, Burgess Hill, UK) on an Opticon 2 detector (Bio-Rad Laboratories, Hemel Hempstead, UK). Reaction mixtures contained 20 ng of cDNA, 1.5 M each primer, 2.5 mM MgCl_2_, and were subjected to a 10 min hot start, followed by 37 cycles of 15 s at 95 °C, 60 s at 55–62 °C and 30 s at 72 °C, with a final 5 min extension. Primer pairs (Invitrogen) were designed using the Primer3 program (http://bioinfo.ut.ee/primer3-0.4.0/). Sequences were TNFα: GTAGCCCACGTCGTAGCAA and GTGGGTGAGGAGCACGTAGT, MCP-1: ACCAGCCAACTCTCACTGAA and ACAGCTTCTTTGGGACACTT, nitric oxide synthase-2 (NOS2): TGACCTGAAAGAGAAAAGGA and TCCAGGGATTCTGGAACATT, 36B4: ACAGCTTCTTTGGGACACTT and ATCTGCTGCATCTGCTTG. The relative abundance of duplicate cDNA aliquots was quantified using a standard curve plotted from amplification of a 10-fold dilution series of DNA generated by conventional PCR from the same primer pairs and gel purified. Results are quoted after normalisation to expression of 36B4, which was unchanged by the treatments. Generation of a single appropriate PCR product was confirmed by melting curve analysis and periodic agarose gel electrophoresis.

### 2.6 Glycogen synthesis

Incorporation of glucose into glycogen by myotubes was measured as previously described (Cazzolli et al., 2001). C2C12 myotubes were serum starved and then 2 μCi D-[U-^14^C]-glucose (NEN/Perkin–Elmer; Waltham, MA, USA) was added per well ±100 μM insulin for one hour. The reaction was terminated by washing ×3 with ice cold PBS. Myotubes were lysed in RIPA buffer at 100 °C for 10 min. Protein content was measured using a bicinchoninic acid assay (Pierce Biotechnology, Rockford, IL). Glycogen was precipitated in ethanol at 4 °C overnight, then at −20 °C for 1 h, before centrifugation at 13,000*g* for 20 min. The pellet was dissolved in water at 60 °C, mixed with scintillant (Ultima Gold, Perkin–Elmer) and counted using a LS6500 beta counter (Beckman Coulter, UK). Results were calculated as pmol/min/mg of protein.

### 2.7 ELISA for secreted peptides

CM was centrifuged at 13,000*g* for 10 min and the cell-free medium aliquoted into collection tubes and stored at −80 °C until analysis. Quantikine colormetrix sandwich ELISAs (R&D Systems, Minneapolis, MN, USA) were used to measure levels of TNFα, IL1β, Chemokine (C-X-C motif) ligand 2 (CXCL2) and monocyte chemoattractant protein (MCP)1. Samples were diluted where necessary and the procedure carried out according to the manufacturer’s instructions. Absorbance was measured on a microplate reader (Tecan, Reading, UK) at 450 nm and corrected by the absorbance at 540 nm.

### 2.8 Arginase assay

J774 cells were treated with FA-containing medium for 16 h overnight and then washed in PBS before being collected and lysed in 150 μl buffer containing 0.1% Triton X-100/10 mM MnCl_2_/25 mM Tris–HCl. Lysates were frozen overnight, defrosted and arginase activity was measured as previously described (Corraliza et al., 1994). 50 μl of lysate was incubated with 50 μl of 0.5 M l-arginine (pH 9.7) for 120 min at 37 °C. The reaction was stopped by the addition of H_2_SO_4_/H_3_PO_4_/H_2_O (1:3:7) and then 25 μl 9% α-Isonitrosopropiophenone in ethanol was added and the mixture heated to 100 °C for 45 min. Samples were then cooled on ice for 30 min before measurement of absorbance at 550 nm.

### 2.9 Griess assay

Inducible nitric oxide synthase (iNOS) activity was assessed by measurement of NO release into the medium. J774 cells were activated with PMA for 3 days and then incubated with phenol red-free DMEM containing BSA-coupled palmitic acid, palmitoleic acid, a combination of both, LPS or vehicle for 16 h. Samples of medium were collected at the end of this experiment, aliquoted into collection tubes and stored at −20 °C before analysis. The Griess assay was performed as a 96 well microplate assay (Molecular Probes, Life Technologies, UK) according to the manufacturer’s instructions and absorbance was read on the plate reader at 548 nm.

### 2.10 Statistics

Statistical analysis of data was performed using GraphPad Prism 6 (GraphPad Software, San Diego, CA, USA). Student’s *t*-test, one-way and two-way ANOVAs were utilised, accompanied as appropriate by *post hoc* testing using Fisher’s least significant difference test. Statistical significance was accepted at *p* < 0.05.

## 3 Results

### 3.1 Insulin-stimulated glycogen synthesis in C2C12 myotubes is impaired in the presence of palmitic acid-treated macrophage-conditioned medium

**Fig. 1:**
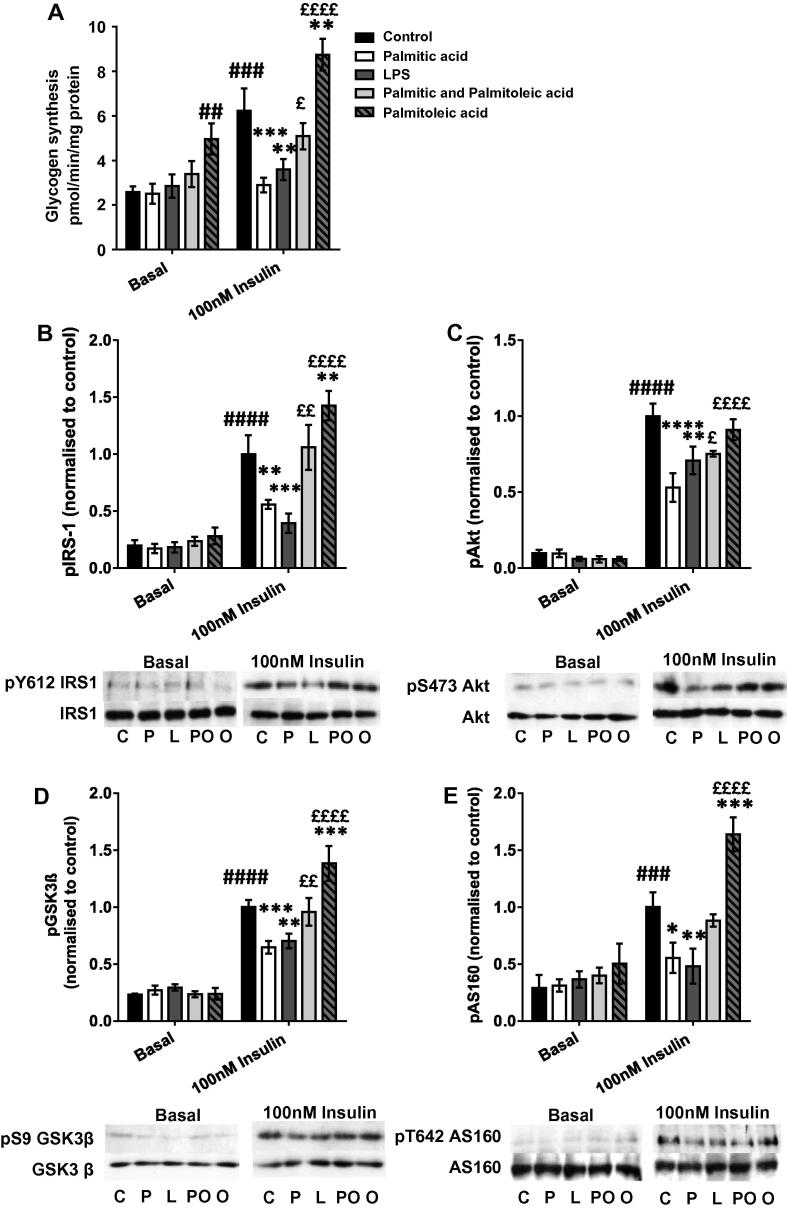
Palmitic acid-treated macrophage-conditioned medium impairs glycogen synthesis and insulin signalling in C2C12 myotubes, while these defects are rescued by palmitoleic acid. C2C12 myotubes were incubated with conditioned medium derived from macrophages treated with LPS, palmitic acid, palmitoleic acid, a combination of the two or vehicle (control group) for 16 h, before being serum starved and (A) incubated with D-[U-^14^C]-glucose tracer ± 100nM insulin to measure glycogen synthesis, or collected in RIPA buffer and SDS–PAGE and immunoblotting conducted to assess phosphorylation and total protein levels of (B) IRS1 (pY612), (C) Akt (pS473), (D) GSK3β (pS9) and (E) AS160 (pT642). Representative blots and summary data (mean ± SEM of 3–6 individual experiments) are shown. All treatment groups were represented on each blot on which bands were quantified, but basal and insulin-stimulated samples are shown separately here for clarity. Total protein levels of all intermediates and β-actin were unchanged by any treatment. Selected *post hoc* significance is shown to simplify interpretation: ^##^*p* < 0.01, ^###^*p* > 0.001 and ^####^*p* < 0.0001 vs. basal control; ^*^p < 0.05, ^**^p < 0.01, ^***^p < 0.001 and ^****^p < 0.0001 vs. control, insulin treated cells; ^£^*p*< 0.05, ^££^*p* < 0.01 and ^££££^*p* < 0.0001 versus palmitate treated, insulin treated cells. C – Control (+ insulin treatment); P – Palmitic acid (+ insulin treatment); L – LPS (+ insulin treatment); PO – Palmitic and palmitoleic acid (+ insulin treatment); O – Palmitoleic acid (+ insulin treatment).

To determine whether CM from macrophages affected glucose disposal or insulin signalling in myotubes, the incorporation of radiolabelled glucose into glycogen and the phosphorylation of insulin signaling intermediates were assessed ± insulin. 100nM insulin caused a 142% increase in glycogen synthesis (*p* = 0.0002), while both positive control LPS-treated macrophage CM (LPS-mac-CM) and palmitic acid-treated macrophage CM (palm-mac-CM) prevented the effect of insulin, with palm-mac-CM causing a 54% reduction (*p* = 0.0005) and LPS-mac-CM a 42% reduction (*p* = 0.0036) versus control CM (Fig. 1A). Insulin stimulation caused substantial increases in phosphorylation of IRS1 (pY612), Akt (pS473), GSK3β (pS9) and TBC1D4/Akt substrate of 160 kDa (AS160; pT642). Both Palm-mac-CM and LPS-mac-CM caused impaired insulin-stimulated phosphorylation of all of these intermediates. LPS-mac-CM caused reductions in phosphorylation of IRS1 (by 61%, *p* = 0.0006), Akt (by 29%, *p* = 0.0026), GSK3β (by 30%, *p* = 0.0039) and AS160 (by 52%, *p* = 0.0043) compared to control-mac-CM (Fig. 1B–E), while palm-mac-CM caused reductions in IRS1 (by 44%, *p* = 0.048), Akt (by 47%, *p* < 0.0001), GSK3β (by 35%, *p* = 0.0009) and AS160 (by 45%, *p* = 0.012) phosphorylation (Fig. 1B–E). There were no effects on total protein levels of these signalling intermediates or on their degree of phosphorylation in the absence of insulin. Thus both LPS- and palm-mac-CM treatment caused impairment in insulin-stimulated glycogen synthesis, associated with impaired insulin signalling from the level of IRS1.

### 3.2 Treatment of macrophages with palmitoleic acid generates conditioned medium that insulin sensitises myotubes

After macrophages were simultaneously incubated with both palmitoleic acid and palmitic acid, the generated CM (comb-mac-CM) did not cause a significant impairment in insulin-stimulated glycogen synthesis in myotubes, as the palm-mac-CM-induced deficit was reduced by 66% (76% higher than palm-mac-CM, *p* = 0.0139; Fig. 1A). Furthermore, CM generated from macrophages treated with palmitoleic acid alone (palmitoleic-mac-CM) increased both basal and insulin-stimulated glycogen synthesis versus the equivalent controls (by 92% and 40%, *p* = 0.008 and *p* = 0.0054 respectively). Similarly, the impairments in insulin-stimulated phosphorylation of PI3K signalling pathway intermediates were largely abrogated when palmitoleate was added to the palmitate-BSA used to treat macrophages. Myotubes incubated with comb-mac-CM showed a 90% increase in pY612-IRS1 compared to palm-mac-CM (*p* = 0.0017), thus restoring phosphorylation to the level of insulin-treated control CM-incubated myotubes (Fig. 1B). Phosphorylation of Akt, GSK3β and AS160 was also variably restored in myotubes (*p* = 0.0026–0.060; Fig. 1C–E). In addition, consistent with the elevated glycogen synthesis, palmitoleic-mac-CM increased pY612-IRS1 (by 43%, *p* = 0.0062; Fig. 1B), pS9-GSK3b (by 39%, *p* = 0.0007; Fig. 1D) and pT642 (by 64%, *p* = 0.0006; Fig. 1E) versus insulin-stimulated control-mac-CM-treated myotubes, while this effect was not detected with respect to pS473-Akt. Total protein levels of each intermediate were again unchanged. Thus, palmitoleic acid treatment of macrophages results in CM that insulin sensitises myotubes and can rescue the defect in glycogen synthesis caused by palm-mac-CM treatment of myotubes.

### 3.3 Palmitic acid and palmitoleic acid-treated macrophage-conditioned media have contrasting effects on inflammatory signalling in C2C12 myotubes

**Fig. 2:**
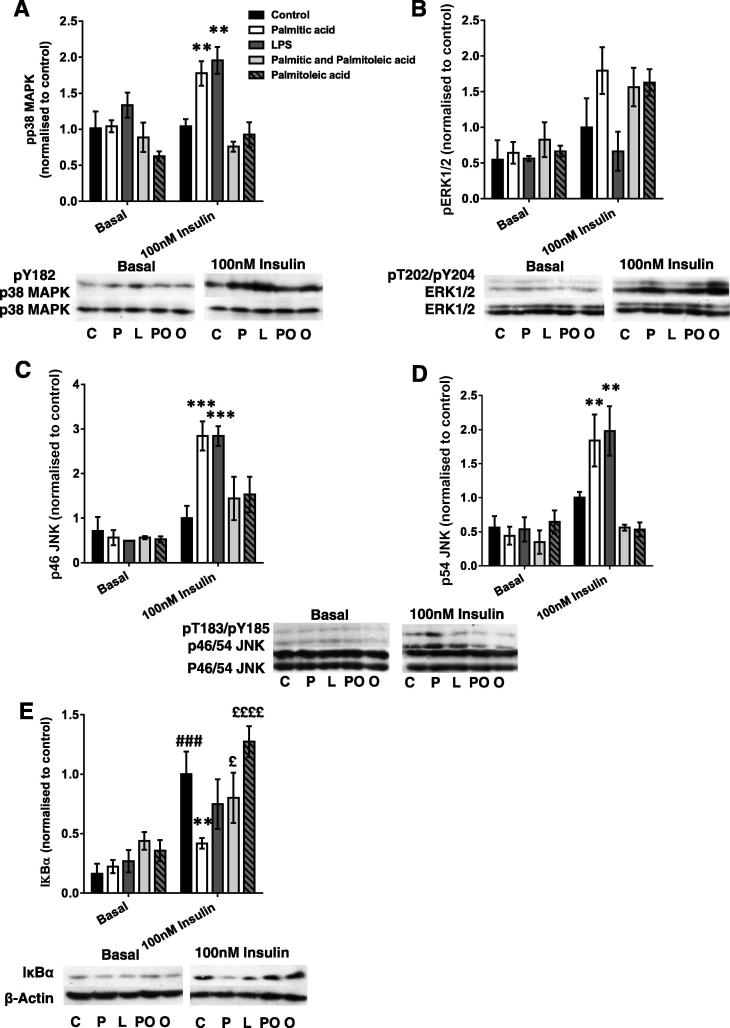
Palmitic acid and palmitoleic acid-treated macrophage-conditioned media have contrasting effects on inflammatory signalling in C2C12 myotubes. C2C12 myotubes were incubated with conditioned medium derived from macrophages treated with LPS, palmitic acid, palmitoleic acid, a combination of the two or vehicle (control group) for 16 h, before being serum starved and lysed in RIPA buffer, and SDS–PAGE and immunoblotting conducted to assess phosphorylation and total protein levels of (A) p38 MAPK (pY182), (B) ERK1/2 (pT202), (C) p46 JNK (pT183/pY185), (D) p54 JNK (pT183/pY185) and E) IκBα. Densitometry was performed and sample blots and summary graphs are shown, representing the mean ± SEM of 3–4 individual experiments. All treatment groups were represented on each blot on which bands were quantified, but basal and insulin-stimulated samples are shown separately here for clarity. Total protein levels of MAPKs and β-Actin were unchanged by the treatments. Selected *post hoc* significance is shown to simplify interpretation: ^*^p < 0.05, ^**^p < 0.01, ^***^p < 0.001 vs. insulin-treated control; ^###^*p* < 0.001 vs. basal control; ^£^*p* < 0.05, and ^££££^*p* < 0.0001 versus palmitate treated, insulin treated cells. C – Control (+ insulin treatment); P –Palmitic acid (+ insulin treatment); L – LPS (+ insulin treatment); PO – Palmitic and palmitoleic acid (+ insulin treatment); O – Palmitoleic acid (+ insulin treatment).

To determine whether palm-mac-CM might activate inflammatory/stress signalling pathways in myotubes, immunoblotting was performed initially for phosphorylated and total MAPKs and Inhibitor κBα (IκBα), which causes cytoplasmic retention of NFκB. Although most of these pathways were activated by palm-mac-CM and LPS-mac-CM, interestingly these effects were only apparent under insulin-stimulated conditions. Both LPS- and palm-mac-CM caused increases in pY182-p38 MAPK (*p* = 0.0003 and *p* = 0.0024; Fig. 2A), accompanied by increases in pY185/pT183-p46 JNK (*p* = 0.0034 and *p* = 0.010) and pY185/T183-p54 JNK (*p* = 0.0001 and *p* = 0.0010; Fig. 2B&C), indicative of greater activation of each kinase. A similar trend was observed for ERK phosphorylation after palm-mac-CM but not LPS-mac-CM treatment (Fig. 2D). Total protein levels of each were unaltered. Insulin treatment increased IκBα (by 512%, *p* = 0.0010) but palm-mac-CM especially reversed this effect (58% decrease, *p* = 0.0041; Fig. 2E), implying activation of the NFκB pathway. Thus multiple pro-inflammatory pathways are activated in myotubes and may be responsible for the palm- and LPS-mac-CM-induced IR. Strikingly, phosphorylation of p38 MAPK, JNK1 and JNK2 were all normalised by palmitoleic-mac-CM and comb-mac-CM (Fig. 2A–C). In contrast, all FA-palm-CMs tended to similarly activate ERK1/2 (not significant by ANOVA; Fig. 2D), suggesting that this MAPK does not mediate the differential effects of SFA and UFA treatment of macrophages on myotube insulin sensitivity. In addition, comb-mac-CM reduced degradation of IκBα compared to palm-mac-CM treatment (91% increase, *p* = 0.049; Fig. 2E). Thus palmitoleic acid treatment of macrophages results in CM that does not generate an inflammatory response involving the MAPK and NFκB signalling pathways in myotubes, in contrast to the effects of palm-mac-CM alone. Furthermore, abrogation of the palm-mac-CM-induced MAPK activation might underpin the insulin-sensitising effects of palmitoleic acid.

### 3.4 Macrophage-conditioned medium-induced defects in glycogen synthesis and insulin signalling are partially restored by pharmacological MAPK inhibition

**Fig. 3:**
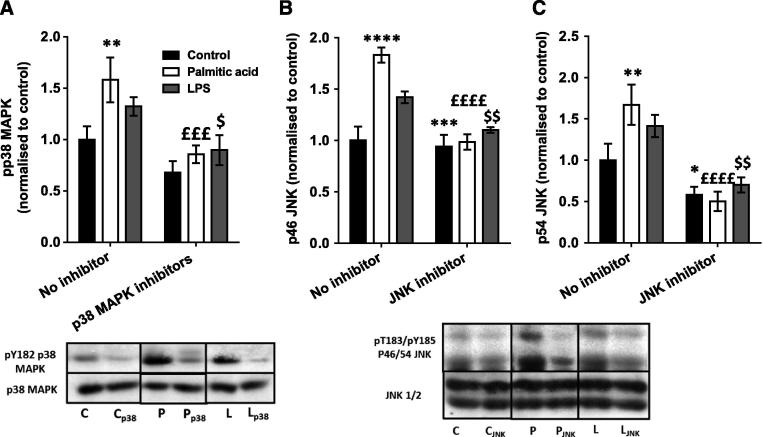
Effects of pharmacological inhibition of MAPKs on conditioned medium-induced changes in MAPK phosphorylation. Conditioned medium from macrophages treated with palmitic acid, LPS or vehicle (control group) was incubated with or without SB205380 (1 μM) and BIRB796 (0.1 μM), JNK V (1 μM) inhibitors or vehicle for 16 h. After cells were serum starved for 1 h and treated with 100nM insulin for 15 min, they were collected in RIPA buffer and phosphorylation and total protein levels of (A) p38 MAPK (pY182), (B) p46 JNK (pT183/pY185) and (C) p54 JNK (pT183/pY185) were assessed by SDS–PAGE and immunoblotting. Summary graphs show the mean ± SEM of 4 individual experiments, accompanied by representative blots. Total protein levels of signalling intermediates and β-Actin were unchanged by the treatments. *Post hoc*: ^*^p < 0.05, ^**^p < 0.01 and ^***^p < 0.001 vs. no inhibitor control; ^£££^*p* < 0.001 vs. no inhibitor palmitic acid; ^$^*p* < 0.05 and ^$$^*p* < 0.01 vs. no inhibitor LPS. C – control conditioned medium (+ insulin treatment); P – palmitic acid-treated macrophage-conditioned medium (+ insulin treatment); L – LPS-treated macrophage-conditioned medium (+ insulin treatment); p38 – SB205380 and BIRB796 inhibitors used; JNK – JNK V inhibitor used.

As previous findings have suggested that activation of the NFκB pathway did not contribute to the generation of IR in muscle *in vivo* (Hommelberg et al., 2011, Polkinghorne et al., 2008), next we aimed to determine whether pharmacological inhibition of the two MAPKs that were activated by SFA and LPS treatment of macrophages could restore the impaired myotube glucose disposal. Glycogen synthesis was measured with or without pre-incubation with the p38 MAPK inhibitors SB203580 (1 μM) and BIRB796 (0.1 μM) (Bain et al., 2007, Kuma et al., 2005) or JNK V inhibitor (1 μM) (Bain et al., 2007). Both palm- and LPS-mac-CM induced increases in pY182-p38 MAPK, pT183/pY185-p46 and p54 JNK, although in the case of LPS-mac-CM, these increases did not reach significance by *post hoc* testing on this occasion, perhaps due to variation in batches of LPS used. Nevertheless, these trends were all abolished by the corresponding inhibitor treatment (Fig. 3A–C), confirming the efficacy of the inhibition. Each MAPK inhibitor alone showed similar but smaller effects when used alone (data not shown).

**Fig. 4:**
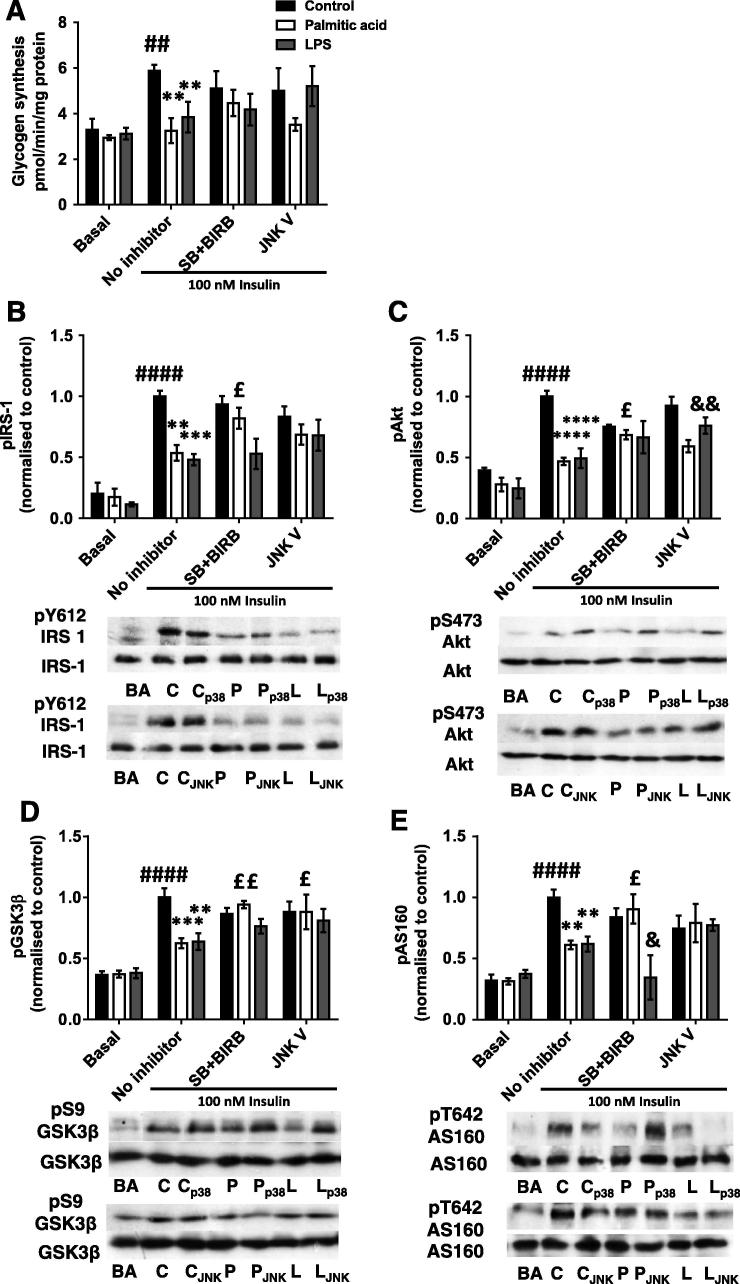
Macrophage-conditioned medium-induced defects in glycogen synthesis and insulin signalling are partially restored by pharmacological MAPK inhibition. C2C12 myotubes were incubated with conditioned medium from macrophages treated with palmitic acid, LPS or control with or without SB205380 (1 μM) and BIRB796 (both 0.1 μM; “SB + BIRB”), JNK V (1 μM) inhibitors or vehicle (“No inhibitor” group) for 16 h, before being serum starved and (A) incubated with D-[U-^14^C]-glucose tracer ± 100nM insulin to measure glycogen synthesis, or collected in RIPA buffer and SDS–PAGE and immunoblotting conducted to assess phosphorylation and total protein levels of (B) IRS-1 (pY612), (C) Akt (pS473), (D) GSK3b (pS9) and (E) AS160 (pT642). Densitometry was performed and these data are shown graphically alongside representative blots. Total protein levels of signalling intermediates and β-Actin were unchanged by the treatments. Data are mean ± SEM of 4–6 individual experiments. Selected *post hoc* significance is shown to simplify interpretation: ^*^p < 0.05, ^**^p < 0.01 ^***^p < 0.001 and ^****^p < 0.0001 vs. no inhibitor insulin-stimulated control; ^£^*p* < 0.05, ^££^*p* < 0.01 vs. no inhibitor palmitic acid; ^&^*p* < 0.05 and ^&&^*p* < 0.01 vs. no inhibitor LPS; ^##^*p* < 0.01 and ^####^*p* < 0.0001 vs. basal (no insulin) control. C – control conditioned medium (+insulin); P – palmitic acid-treated macrophage-conditioned medium (+ insulin): L – LPS-treated macrophage-conditioned medium (+ insulin). BA – basal (no insulin); p38 – SB205380 and BIRB796 inhibitors used; JNK – JNK V inhibitor used.

The effects of MAPK and JNK inhibition on myotube glycogen synthesis and signalling were investigated next. Inhibition of neither of these kinases had effects in non-insulin treated cells, as would be expected based on the data presented in Fig. 2 (data not shown). However, pre-incubation with SB203580 and BIRB796 together had dual effects on insulin-stimulated glycogen synthesis. The 45% reduction in insulin-stimulated glycogen synthesis in palm-mac-CM-treated cells (*p* = 0.0038) was nearly abolished by inhibitor treatment, with a lesser effect in LPS-mac-CM-treated myotubes (Fig. 4A). This was apparently contributed to by both ameliorations of the effects of the mac-CM treatments and an inhibitor-induced reduction in control-mac-CM insulin-stimulated glycogen synthesis. Similarly, p38 MAPK inhibition caused a 53% increase in insulin-stimulated pY612-IRS1 versus that in palm-mac-CM-treated myotubes (*p* = 0.0183; Fig. 4B), substantially restoring the defect. This was accompanied by partial restorations of pS473-Akt (increased by 46%, *p* = 0.025), pS9-GSK3β (increased by 51%, *p* = 0.0028) and pT642-AS160 (increased by 48%, *p* = 0.032) (Fig. 4C-E), with p38 MAPK inhibition also limiting the effects of insulin on phosphorylation of PI3K signalling intermediates in control cells. Interestingly, the LPS-mac-CM-induced defects in insulin signalling were not ameliorated by p38 MAPK inhibition (Fig. 4B-E). Indeed, there was a further 44% reduction in pT642-AS160 compared to LPS-mac-CM treated myotubes (*p* = 0.044; Fig. 4B).

JNK inhibition appeared to partially restore the palm-mac-CM-induced defects in PI3K signalling in insulin-stimulated cells (Fig. 4B-E), although these effects were of lower magnitude than that induced by p38 MAPK inhibition and did not appear to be reflected in improved glycogen synthesis (Fig. 4A). However, inhibition of JNK did cause a 41% increase in pS9-GSK3β versus palm-mac-CM (*p* = 0.013; Fig. 4D). Instead, JNK inhibition was more effective in restoring the defects induced in glycogen synthesis and phosphorylation of signalling intermediates caused by LPS-mac-CM, in particular with regard to pS473-Akt (54% increase over LPS-mac-CM alone; *p* = 0.0067; Fig. 4C), which may be the result of a different secretory profile of macrophages induced by LPS versus palmitic acid. Nevertheless, it appears that JNK is less important than p38 MAPK in the palm-CM-induced defect in myotube glycogen synthesis.

### 3.5 The palmitic acid-treated macrophage CM-induced defect in myotube PI3K signalling is also partially restored by siRNA-induced silencing of p38α

**Fig. 5:**
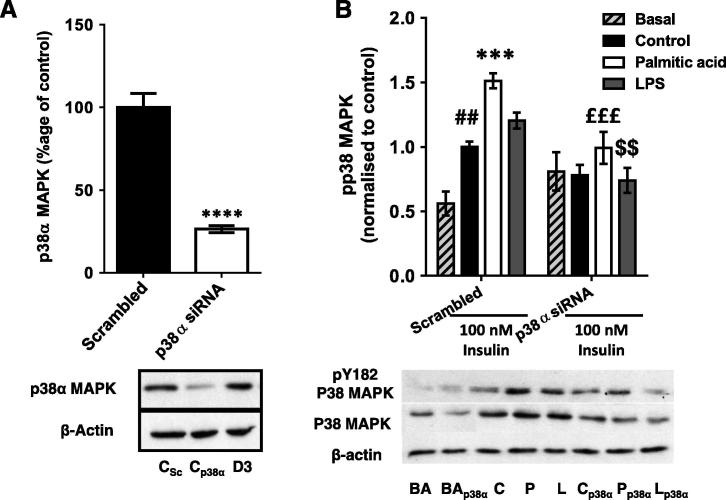
Effects of siRNA-mediated p38 MAPKα silencing on conditioned medium-induced changes in p38 MAPK phosphorylation and protein levels in C2C12 myotubes. Western blots were generated using lysates derived from myotubes incubated with a siRNA pool targeting p38 MAPKα or a nonsense (scrambled) control for 72 h. (A) Silencing of p38α MAPK protein in 100nM insulin-treated C2C12 myotubes (mean 74% reduction). (B) C2C12 myotubes were incubated with conditioned medium generated by macrophages treated with palmitic acid, LPS or vehicle (control group) ± p38α siRNA pool for 16 h. Cells were serum starved for 1 h and then treated ± 100nM insulin for 15 min. Total phosphorylation and total protein levels of p38 MAPK (all isoforms) were assessed. Summary graphs show the mean ± SEM of 4 individual experiments, accompanied by representative blots. Selected *post hoc* significance is shown to simplify interpretation: ^##^*p* < 0.01 vs. scrambled siRNA, basal ^***^p < 0.001 and ^****^p < 0.0001 vs. scrambled siRNA, insulin-treated; ^£££^*p* < 0.001 vs. scrambled siRNA palmitic acid; ^$$^*p* < 0.01 vs. scrambled siRNA LPS. C – control conditioned medium (+ insulin treatment); P – palmitic acid-treated macrophage-conditioned medium (+ insulin treatment); L – LPS-treated macrophage-conditioned medium (+ insulin treatment). BA – basal (no insulin); p38α – p38 MAPKα siRNA-treated; D3 – transfection reagent-treated only.

As pharmacological inhibition of p38 MAPK had more striking effects than that of JNK and these effects were more obvious with regard to insulin signalling than glycogen synthesis, we sought to corroborate this finding by investigating the effects of siRNA-mediated silencing of the principal pro-inflammatory p38 MAPK isoform in skeletal muscle (p38α) (Kuma et al., 2005) on myotube insulin signalling. A 74% reduction in p38α protein was obtained (*p* < 0.0001; Fig. 5A) and this was sufficient to reduce collective phosphorylation of all the p38 MAPK isoforms. Specifically, palm-mac-CM had an additive effect with insulin (Antonescu et al., 2005) to increase pY182-p38 MAPK, but both these increases were abolished by siRNA. A 34% decrease in total pY182-p38 MAPK occurred (*p* = 0.0007), reducing this to the level measured in control myotubes (Fig. 5B), a trend that was replicated in LPS and control-CM-incubated myotubes, again demonstrating a dual effect of manipulating p38 MAPK on insulin signalling. Similarly, total p38 MAPK protein was reduced by a mean of 45% across all groups (ANOVA *p* = 0.0003; Fig. 5B), reflecting residual expression of other MAPK isoforms.

**Fig. 6:**
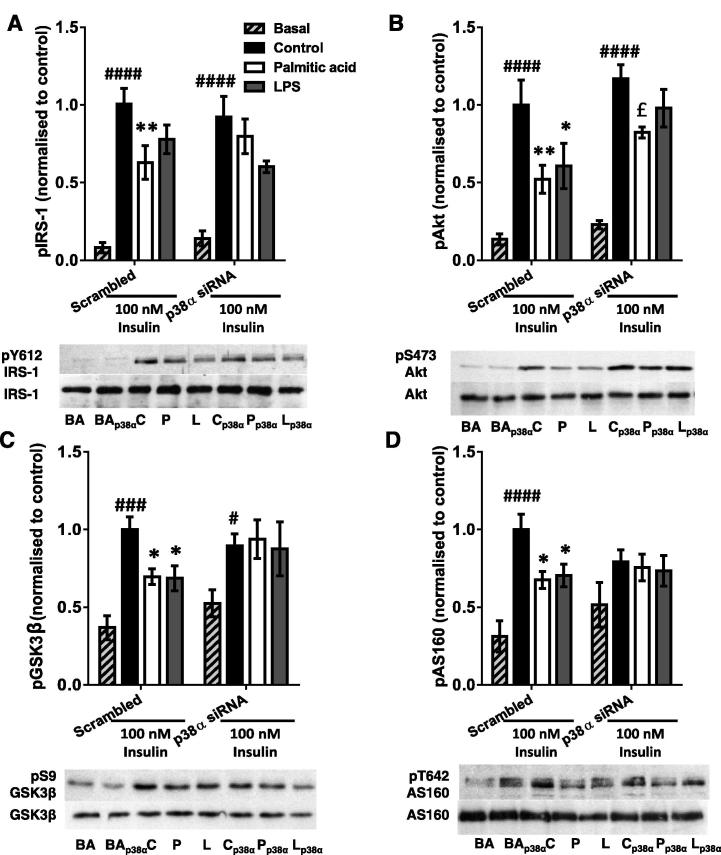
The palmitic acid-treated macrophage-CM-induced defect in myotube PI3K signalling is partially restored by siRNA-induced silencing of p38α. Conditioned medium from macrophages treated with palmitic acid, LPS or vehicle (control group) was used to incubate C2C12 myotubes ± a p38 MAPKα siRNA pool for 16 h. Cells were serum starved for 1 h and treated ± 100nM insulin for 15 min. Phosphorylation and protein levels of (A) IRS-1 (pY612), (B) Akt (pS473), (C) GSK3β (pS9) and (D) AS160 (pT642) were assessed by western blotting of lysates. Densitometry was performed on blots and summarised as graphs showing the mean ± SEM of 4 individual experiments, alongside representative blots. Total protein levels were unchanged by any treatment. Selected *post hoc* significance is shown to simplify interpretation: ^*^p < 0.05 and ^**^p < 0.01 vs. scrambled siRNA insulin-treated control; ^£^*p* < 0.05 vs. scrambled siRNA palmitic acid; ^#^*p* < 0.05, ^###^*p* < 0.001 and ^####^*p* < 0.0001 vs. basal (no insulin) sample from the same siRNA treatment group. C – control conditioned medium (+ insulin); P – palmitic acid-treated macrophage-conditioned medium (+ insulin); L – LPS-treated macrophage-conditioned medium (+ insulin). BA – basal (no insulin); p38α – p38 MAPKα siRNA-treated.

The siRNA-mediated reduction in p38 MAPKα ameliorated most of the observed signalling defects to varying extents, as indicated by the abolition of the significant differences in phosphorylation between insulin-treated control and palm- or LPS-mac-CM-treated myotubes (Fig. 6A-D). Most clearly, there was a significant increase in phosphorylation of Akt after palm-mac-CM treatment (58%; *p* = 0.034; Fig. 6B) and a similar trend for GSK3β phosphorylation (35% increase, *p* = 0.10; Fig. 6C), confirming that this was not purely due to an inhibition of control insulin-stimulated phosphorylation, although this likely contributes, as with the effects of pharmacological inhibition. In particular, this could provide an explanation for the changes observed in AS160 phosphorylation (Fig. 6D), or indeed this could be the result of effects upon activity of an alternative upstream kinase, such as AMP-activated protein kinase (AMPK) (Thong et al., 2007). However, the data are broadly consistent with those generated using p38 MAPK inhibitors, confirming a role for p38 MAPK in the mechanism.

### 3.6 Macrophages treated with palmitic acid show a pro-inflammatory polarisation that is abrogated by co-incubation with palmitoleic acid

**Fig. 7:**
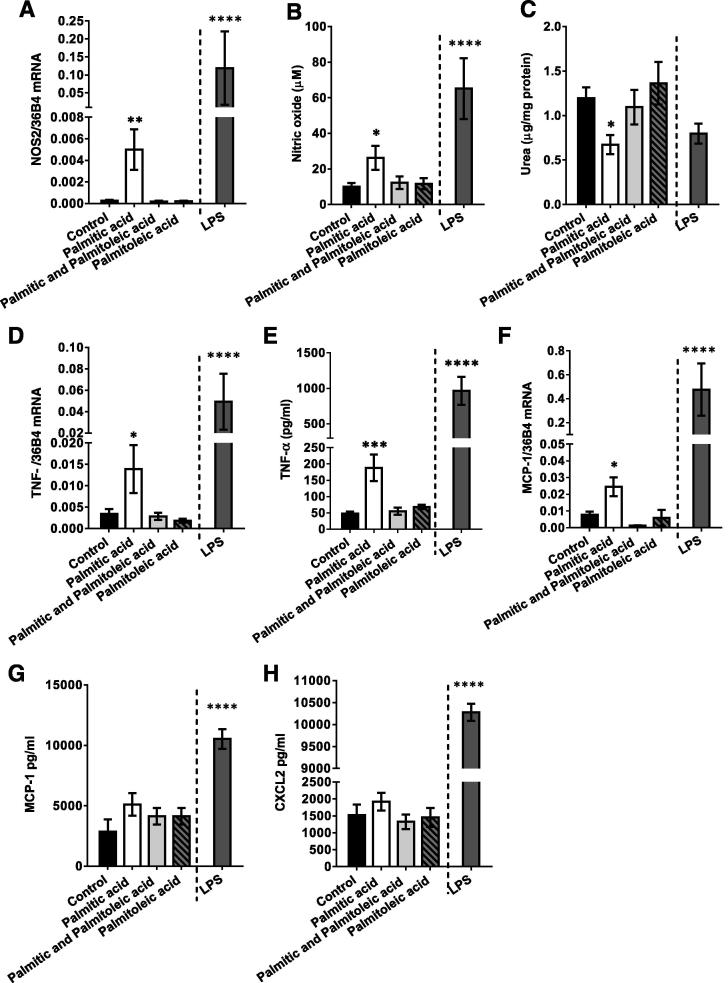
Macrophages treated with palmitic acid show a pro-inflammatory polarisation that is abrogated by the addition of palmitoleic acid. J774 Macrophages were incubated with 0.75 mM palmitic acid, 0.75 mM palmitoleic acid, a combination of both, LPS, or vehicle (control group) for 8 h, washed with PBS and then conditioned medium (CM) generated for 16 h. After this period, CM and macrophages were collected and (A) macrophage mRNA expression of NOS2 was measured, (B) content of nitric oxide (μM) in culture medium was measured using the Griess assay, (C) arginase activity was assessed based on the amount of urea (μg) produced per mg of protein, (D) TNFα mRNA and (E) TNFα peptide were measured in macrophages/CM respectively by real-time PCR/ELISA, (F) MCP1 mRNA and G) MCP1 peptide were measured in macrophages/CM respectively and (H) CXCL2 peptide was measured in CM by ELISA. Values are the mean ± SEM of 5–6 individual experiments. *Post hoc*: ^*^p < 0.05, ^***^p < 0.001 and ^****^p < 0.0001 vs. control.

We next investigated the effects of each FA treatment on macrophage polarisation and secretion of potential mediators of the effects observed in the myotubes. Macrophage activation is classically defined as either M1 (pro-inflammatory) or M2 (anti-inflammatory), according to cellular iNOS and arginase activities (Lumeng et al., 2007a, Lumeng et al., 2007b), therefore NOS2 expression in, NO release by and arginase activity of treated macrophages were measured. Macrophages treated with LPS demonstrated robust respective 44 and 5.6-fold increases in NOS2 and NO release over control (*p* < 0.0001 and *p* = 0.0002; Fig. 7A and B). However, in addition, palmitic acid-treated macrophages also showed an 18-fold increase in NOS2 mRNA (*p* = 0.0074) and produced 164% more NO than control macrophages (*p* = 0.012), while palmitoleic acid and the combined treatment did not affect NOS2 mRNA or NO release. Palmitic acid-treated macrophages also produced 44% less urea per mg of protein than control macrophages (*p* = 0.034), although the reduction caused by LPS treatment was not significant (Fig. 7C). In contrast, palmitoleic acid or palmitic/palmitoleic acid-treated cells produced a similar amount of urea to control cells. Thus palmitic acid-treated macrophages demonstrate a classical pro-inflammatory M1 phenotype on the basis of these two assays, while this shift is prevented in the presence of palmitoleic acid.

M1-activated macrophages secrete a range of pro-inflammatory cytokines/chemokines that activate inflammatory pathways in adjacent cells, therefore the levels of 2 pro-inflammatory cytokines and 2 pro-inflammatory chemokines that could be implicated in muscle IR (De Paepe et al., 2012, Hotamisligil et al., 1993, Sell et al., 2006, Tardif et al., 2011) were measured in mac-CM. LPS treatment of macrophages caused the expected substantial increases in production of TNFα, MCP1 and CXCL2 (all *p* < 0.0001; Fig. 7D-H). However, there were also respective 308 and 296% increases in TNFα macrophage mRNA and peptide in palm-mac-CM versus control (*p* = 0.0101 and *p* = 0.001; Fig. 7D and E), changes that were normalised by concurrent palmitoleic acid incubation. MCP1 mRNA was increased by 213% in macrophages (*p* = 0.0074; Fig. 7F) but the increase in peptide concentration in palm-mac-CM did not reach significance (79% increase, *p* = 0.15; Fig. 7G), and this was not substantially affected by addition of palmitoleic acid. No effects on CXCL2 secretion were noted (Fig. 7H), while IL1β levels were below the level of detection for treatment groups other than LPS-mac-CM (data not shown). This suggests that neither CXCL2 nor IL1β are likely to be responsible for the effects of CM in myotubes, while the evidence for a role of MCP1 is equivocal.

### 3.7 Addition of a TNFα blocking antibody leads to partial restoration of the palmitic acid-treated macrophage CM-induced defect in myotube insulin signalling

As palm-mac-CM contained elevated levels of TNFα and this increase was prevented by co-incubation with palmitoleic acid, we aimed to establish whether TNFα might be responsible for the induction of myotube IR by palm-mac-CM. Preliminary experiments confirmed that 2.5–100 ng/ml recombinant TNFα decreased pS473-Akt in myotubes, confirming that TNFα is capable of having this effect, while the TNFα blocking antibody did not affect Akt phosphorylation. In addition, 1 ng/ml recombinant TNFα increased pY185-JNK1/pT183-JNK2, but this was prevented by 10 μg/ml blocking antibody, while there was no activation of this kinase by the blocking antibody alone (data not shown). Therefore, in further experiments 10 μg/ml blocking antibody was added to palm-mac-CM and LPS-mac-CM prior to their addition to myotube cultures.

**Fig. 8:**
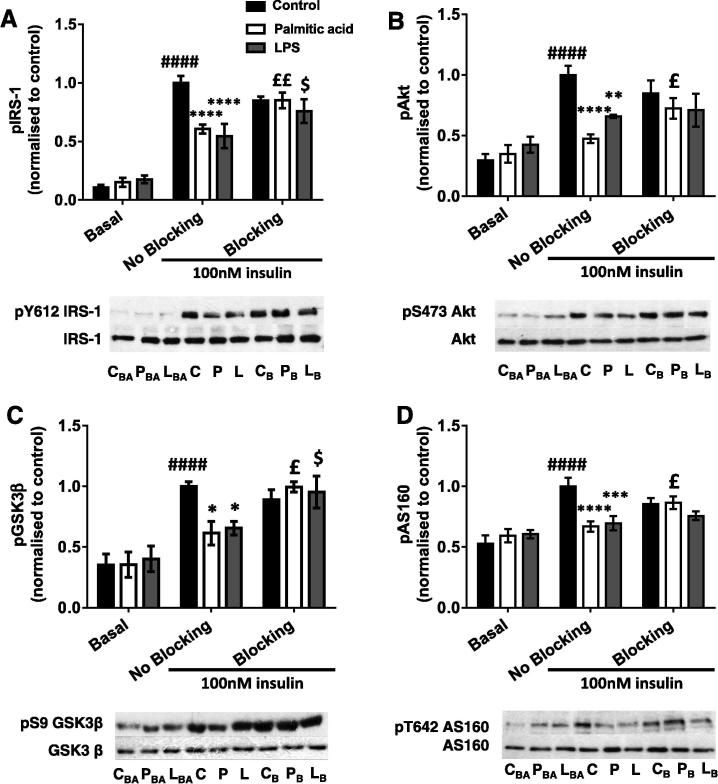
Addition of a TNFα blocking antibody leads to partial restoration of the palmitic acid-treated macrophage CM-induced defect in myotube insulin signalling. C2C12 myotubes were incubated with conditioned medium derived from macrophages treated with palmitic acid, LPS or vehicle (No blocking group), with or without TNFα blocking antibody (10 μg/ml) for 16 h, before being serum starved and lysed in RIPA buffer, and SDS–PAGE and immunoblotting conducted to assess phosphorylation and total protein levels of (A) IRS1 (pY612), (B) Akt (pS473), (C) GSK3β (pS9) and (D) AS160 (pT642). Representative blots and summary data (mean ± SEM of 6 individual experiments) are shown. Protein expression levels of all intermediates were unchanged by any treatment. Selected *post hoc* significance is shown to simplify interpretation: ^*^p < 0.05, ^**^p < 0.01, ^***^p < 0.001 ^****^p < 0.0001 vs. insulin-treated control; ^£^*p* < 0.05; ^££^*p* < 0.01 vs. No Blocking palmitic acid; ^$^*p* < 0.05 vs No Blocking LPS; ^####^*p* < 0.0001 vs. Basal control. B – Basal (no insulin) C – Control, insulin-treated P – Palmitic acid, insulin treated, L – LPS, insulin treated. _BA_ – Basal, _B_ – Blocking antibody present.

Insulin caused the expected increases in phosphorylation of all signalling intermediates in control-mac-CM myotubes (all *p* < 0.0001), while the addition of blocking antibody had no significant effect, although there did tend to be small reductions in each (Fig. 8A–D). The expected reductions in phosphorylation of signalling intermediates caused by palm-mac-CM and LPS-mac-CM (*p* = 0.012 to *p* < 0.0001; Fig. 8A-D) were attenuated in the presence of blocking antibody. There were 30–62% increases in pY612-IRS-1, pS473-Akt, pS9-GSK3β and pT642-AS160 (range *p* = 0.0079 to *p* = 0.0271; Fig. 8A-D) in myotubes incubated with palm-mac-CM, which were sufficient to restore phosphorylation of each intermediate to near normal levels. In addition, blocking antibody lead to a 30% increase in pY612-IRS1 (*p* = 0.019; Fig. 8A) and a 29% increase in pS9-GSK3β (*p* = 0.042; Fig. 8C) when added to LPS-mac-CM, while Akt and AS160 phosphorylation did not change significantly. Thus TNFα is a major mediator of the induction of myotube IR by palm-mac-CM, while LPS-mac-CM likely has effects mediated through other cytokines in addition to TNFα.

## 4 Discussion

In this study, we have utilised a model involving macrophage treatment with an SFA, a UFA or a combination of these and application of the CM generated to differentiated C2C12 myotubes in culture to determine whether and how increased local infiltration of macrophages into skeletal muscle during obesity ((Fink et al., 2013, Hevener et al., 2007, Nguyen et al., 2007); NAT, CWJ and MEC, unpublished data) might impact upon local insulin sensitivity and the differential effects of FA types in this context. The principal findings were that palmitate treatment induces an M1-type polarisation of macrophages and increased secretion of TNFα, which causes activation of inflammatory/stress signalling pathways in myotubes and thus inhibition of insulin-stimulated glucose uptake and incorporation into glycogen, while treatment of macrophages with palmitoleic acid results in myotube insulin sensitisation and is capable of preventing the effects of palmitate in this model. Importantly, we verified that there was no detectable carry-over of FAs into the CM after cell washing and that there was no effect of control-mac-CM on myotube glycogen synthesis versus medium that had not been incubated with macrophages (data not shown).

Our data are generally consistent with a recent study in which CM generated by palmitate treatment of RAW macrophages caused reductions in glucose uptake and insulin-stimulated signalling in GLUT4-overexpressing L6 myoblasts (Samokhvalov et al., 2008). However, arguably our model utilising unmodified differentiated myotubes and macrophages from the same species (mouse) reflects the *in vivo* situation more closely. Interestingly, this previous study found that LPS-mac-CM actually had positive effects on insulin signalling, while we found its effects to be similar to those of palm-mac-CM, perhaps due to these differences in the models used or contrasting secretory profiles of the macrophage lines. Instead, our data are consistent with the proposal that both LPS and SFAs activate macrophages through binding to Toll-like Receptor-4 (Hwang, 2001, Lee et al., 2001, Nguyen et al., 2007). Our findings also clearly implicate a role for TNFα-induced activation of p38 MAPK, a mechanism that has been shown to impair insulin sensitivity in muscle cells before (de Alvaro et al., 2004). The effect of TNFα on myotubes involved activation of IKK and thus NFκB downstream of p38 MAPK in that study, which is consistent with the reduced levels of IκBα we observed. The Klip group have identified a role for novel PKC isoform activation in causing mac-CM-induced effects in myoblasts (Kewalramani et al., 2011). Since previously the effects of hyperglycaemia in monocytes have been ascribed to conventional PKC-mediated p38 MAPK activation (Devaraj et al., 2005), it is possible that novel PKC activation may occur upstream of p38 MAPK and IKK activation in our model.

Numerous publications have suggested that stress kinases such as JNK, p38 MAPK and IKK serine phosphorylate IRS1, leading to reduced tyrosine phosphorylation and thus reduced downstream signalling to glucose disposal (Aguirre et al., 2002, Fujishiro et al., 2003, Hemi et al., 2011, Hirosumi et al., 2002, Yuan et al., 2001), although IRS1-independent mechanisms have also been proposed (Cleasby et al., 2007, Hoehn et al., 2008). We found that activating phosphorylation of both JNK and p38 MAPK were increased in myotubes, whereas this trend did not reach significance in the case of ERK, as was found in a human cell co-culture model (Varma et al., 2009). Pharmacological inhibition of p38 MAPK lead to partial restoration of palm-mac-CM-induced defects in glucose utilisation and signalling, data that were mostly recapitulated using p38 MAPKα siRNA. However, the abolition of the palm-mac-CM effects was due at least in part to the dual effects of p38 MAPK inhibition on insulin signalling: not only can p38 MAPK impair glucose utilisation (de Alvaro et al., 2004), but there is some evidence that it is also required for full insulin stimulated glucose uptake and PI3K signalling (Antonescu et al., 2005). The lesser effects of the siRNA-mediated gene silencing may be ascribed to the <80% reduction in p38α MAPK produced and/or the targeting of only the α-isoform, although this is the principal pro-inflammatory isoform in muscle (Boppart et al., 2000). Thus, targeting of p38 MAPK in muscle may be of therapeutic benefit for obesity-associated IR. In support of this, pharmacological inhibition of p38 MAPK improved basal glucose disposal in cultured adipocytes (Carlson and Rondinone, 2005), reduced obesity and whole body IR induced by high fat diet-feeding in rodents (Maekawa et al., 2010) and alleviated oxidative stress-associated IR in soleus muscle (Diamond-Stanic et al., 2011). In contrast, the pharmacological attenuation of JNK activity did not significantly prevent the palm-mac-CM-induced defects. Instead, this treatment showed some efficacy in ameliorating the similar defects induced by LPS-mac-CM, suggesting that LPS and palmitate treatment of macrophages may result in the release of a different range of soluble mediators that induce muscle IR through activation of alternative MAPKs. Indeed, Samokhvalov et al. showed that LPS treatment of macrophages elicited secretion of the anti-inflammatory cytokine IL-10, which might account for the similar effects of LPS-mac-CM and palm-mac-CM on myotubes, despite the far larger macrophage TNFα secretion induced by the former treatment.

Of the four cytokines/chemokines measured here, only TNFα secretion was significantly increased by palmitic acid treatment, but the importance of this finding was confirmed by repeating myotube incubation with palm-mac-CM in the presence of a TNF-α blocking antibody. This cytokine also played a role in mediating the effects of palm-mac-CM on L6 myoblasts (Samokhvalov et al., 2008), while TNFα is generally well-established as a mediator of IR, as infusion leads to reduced whole body insulin-mediated glucose uptake in humans (Plomgaard et al., 2005), while knockout of TNFα or its receptors protect mice from obesity (Hotamisligil et al., 1994, Uysal et al., 1997). In support of our proposed cellular mechanism, TNFα has been shown to induce p38 MAPK-dependent serine phosphorylation of both the insulin receptor and IRS1, reducing insulin-stimulated PI3K signalling, glucose uptake and GLUT4 translocation in myotubes (de Alvaro et al., 2004). However, an additional role for MCP1 (Sell et al., 2006) or other cytokines/chemokines in the mechanism has not been ruled out.

We have shown that macrophages treated with palmitoleic acid alone generated CM that was insulin sensitising, as well as increasing basal glycogen synthesis, while addition of palmitoleic acid to a palmitic acid incubation is also capable of preventing the IR associated with the palm-mac-CM-induced activation of MAPKs, suggesting an additional mechanism whereby UFAs have insulin sensitising effects in skeletal muscle. These effects may be mediated either by anti-inflammatory factors secreted by the macrophages that have insulin sensitising effects in myotubes or by modifying activation of intracellular signalling pathways in macrophages, although further work will be needed to assess these possibilities. Similar effects of palmitate and palmitoleate on bone marrow-derived macrophage M1/M2 polarisation have been observed previously (Prieur et al., 2011), while UFAs have been shown to prevent SFA-induced NFκB activation (Lee et al., 2001) in macrophages.

Palmitate and palmitoleate have distinct direct effects upon insulin-stimulated glucose disposal in L6 myotubes (Dimopoulos et al., 2006), but the effects of palmitoleate were not shown to be mediated through a protective effect on insulin signalling previously. However, others have shown that oleate protects against the effects of palmitate by reducing the impairment in PI3K signalling (Coll et al., 2008, Gao et al., 2009) and through anti-inflammatory effects (Coll et al., 2008), including prevention of MAPK activation (Kadotani et al., 2009). This is likely mediated through reduced synthesis of ceramide and/or diacylglycerol in palmitate-treated myotubes (Chavez and Summers, 2003), rather than accumulation of the more inert triacylglycerol and this mechanism may also result in increased pro-inflammatory cytokine production by macrophages (Schilling et al., 2013).

Our data imply that palmitoleate has dual effects in muscle when mediated through the altered secretory milieu generated by macrophage treatment, on both basal and insulin-stimulated glucose disposal, associated with activation of the PI3K signalling pathway. Importantly, the beneficial effects of palmitoleic acid have also been demonstrated *in vivo*, as IR is improved by the feeding of a diet high in UFAs (Tardif et al., 2011) and by infusion of palmitoleate (Cao et al., 2008) in rodents. Indeed, Cao et al. proposed that palmitoleate represented a specific novel lipid signal, or “lipokine”, which is not found in large amounts in the diet, but instead is released in more substantial amounts from adipose tissue and regulates muscle metabolism (Cao et al., 2008, Pinnick et al., 2012).

## 5 Conclusions

We have demonstrated that IR in muscle may at least in part result from the effects of SFAs on local macrophages, causing release of pro-inflammatory cytokines, paracrine activation of stress signalling in adjacent muscle fibres and thus impaired insulin-stimulated glucose incorporation into glycogen. This phenomenon is likely to be of most significance when increased numbers of macrophages accumulate in muscle during obesity. Further work must aim to establish whether this represents a quantitatively important mechanism of generation of muscle IR *in vivo*. The presence of palmitoleate is sufficient to prevent these effects, although it is unclear whether this reflects a universal property of UFAs or a “lipokine” effect. Nevertheless, these findings serve to reinforce the likely health benefits of a diet rich in UFAs rather than SFAs and suggest that therapeutic targeting of TNFα action and p38 MAPK activity remain potential approaches for the treatment of T2D.
